# A simple instrument to find spatiotemporal overlap of optical/X-ray light at free-electron lasers^1^

**DOI:** 10.1107/S1600577519002248

**Published:** 2019-04-05

**Authors:** Takahiro Sato, James M. Glownia, Matthiew R. Ware, Matthieu Chollet, Silke Nelson, Diling Zhu

**Affiliations:** a Linac Coherent Light Source, SLAC National Accelerator Laboratory, 2575 Sand Hill Road, Menlo Park, CA 94022, USA; b PULSE Institute, SLAC National Accelerator Laboratory, 2575 Sand Hill Road, Menlo Park, CA 94025, USA

**Keywords:** X-ray free-electron lasers, optical laser pulses, pump-probe experiments, spatial overlap, temporal overlap

## Abstract

A compact and robust diagnostic to determine spatial and temporal overlap between X-ray free-electron laser and optical laser pulses was developed and evaluated using monochromatic X-rays from the Linac Coherent Light Source. It was used to determine temporal overlap with a resolution of ∼10 fs, despite the large pulse energy fluctuations of the monochromatic X-ray pulses, and covers a wide optical wavelength range from ultraviolet to near-infrared with a single configuration.

## Abstract 2

Development and evaluation of a spatial and temporal determination diagnostic for X-ray free-electron lasers.

## 1. Introduction   

X-ray free-electron laser (XFEL) sources have generated hard X-ray pulses with high peak power (∼10 GW), ultrashort pulse duration (∼10 fs) and high spatial coherence (Emma *et al.*, 2010 ▸; Ishikawa *et al.*, 2012 ▸; Kang *et al.*, 2017 ▸). The capabilities of XFEL sources enable scientific investigations at the frontiers of both spatial and temporal resolutions (Bostedt *et al.*, 2016 ▸). In particular, pump–probe measurements that combine ultrafast optical laser and XFEL pulses are one of the most important techniques for observing ultrafast phenomena. One of the key procedures in time-resolved experiments is the establishment of spatial and temporal overlap, *i.e.* ‘time zero’, between the ultrafast optical laser and XFEL pulses with micrometre and sub-picosecond scale accuracy. In order to establish spatial overlap, scintillation materials such as Ce:YAG are often utilized to image the spatial locations and profiles of both beams. To find time zero, several techniques have been developed including ultrafast melting, optical coherent phonons induced by the optical laser, and the optical transmission change of Ce:YAG, GaAs or other materials induced by the intense X-ray pulses, which are pump–probe experiments by themselves after the spatial overlap has been established. For instance, the X-ray detection of optical coherent phonons of bismuth is an established technique to determine timing overlap (Fritz *et al.*, 2007 ▸). Using this technique, however, we need to first measure coarse timing by using a fast photodiode with an accuracy of typically 10–20 ps using a fast oscilloscope, then locate the Bragg peak of the bismuth thin film via a rocking-curve scan, and finally measure the probe delay dependent diffraction intensity through accumulating sufficient statistics with both longer (−20 to 20 ps) and shorter (−2 to 2 ps) range time-delay scans with normalization. Another timing method is to observe the optical transmission change of a sample originating from photo-absorption of intense X-rays and its core electron excitation. The primary electrons excited through the photo-absorption process relax through complex secondary processes such as Auger decays and electron–electron scattering that generate a huge number of carriers in the material and subsequently change the real and imaginary parts of the refractive index (Harmand *et al.*, 2012 ▸; Medvedev *et al.*, 2013 ▸; Medvedev, 2015 ▸; Sanchez-Gonzalez *et al.*, 2017 ▸; Mecseki *et al.*, 2018 ▸). This is a promising and simple technique to find temporal overlap without any special analysis and has been applied to arrival timing diagnostics in hard X-ray regions (Harmand *et al.*, 2013 ▸; Sato *et al.*, 2015 ▸; Katayama *et al.*, 2016 ▸; Chollet *et al.*, 2015 ▸). Generally, the focused optical laser and focused or unfocused X-rays co-propagate through a sample and the transmitted laser pulse energy is monitored as a function of time. The key parameters influencing the magnitude of the change in transmission are spatial matching between the two beams, and the X-ray photon flux. With the full energy (few mJ) pink beam of the XFEL, it is easy to observe a large optical transmission change even with a large spatial mismatch between the optical and X-ray beams simply because of the huge magnitude of the transmission drop. We can also observe a decent change in transmission in the sample with the unfocused X-ray beam because of the large number of charge carriers generated. However, 70%–80% of experiments at the X-ray pump–probe (XPP) instrument at the Linac Coherent Light Source (LCLS) use monochromatic X-rays. The monochromatic X-rays have µJ pulse energies and larger intensity fluctuations compared with the full pink beam because of self-amplified spontaneous emission (SASE) characteristics. In addition, the low pulse energies necessitate the use of a tight X-ray focus that is much smaller than the optical laser diameter at the sample position to obtain an appreciable change in the sample opacity, which is not optimum for an X-ray pump, optical probe experiment to detect the transmission change. These conditions combined make it much more difficult to locate temporal overlap using the transmission change. Furthermore, future experiments using the lower pulse energy associated with high repetition rate, narrow bandwidth X-ray pulses, or X-ray split and delay optics could make these conditions even more difficult.

Here we describe the development and evaluation of an alternative diagnostic, ‘t0-finder’, to increase signal contrast by combining a thin diffusive scintillating material and an imaging device. This diagnostic has a capability of determining spatial overlap and time zero, which is nearly background-free by using the correlation methodology. The measurable time delays cover from several tens of femtoseconds to the sub-microsecond range, and we benchmarked this technique by performing a cross-correlation measurement of transmission changes using monochromatic X-rays and optical laser wavelengths covering from the UV to near-infrared.

## 2. Experimental   

To evaluate the t0-finder we utilized the XPP instrument at the LCLS (Chollet *et al.*, 2015 ▸) and performed commissioning of the t0-finder using four different optical laser wavelengths. The central photon energy of the X-rays was 9.5 keV with bandwidth of ∼30 eV. The X-rays were monochromized by a diamond (111) monochromator with bandwidth of 0.5 eV (Zhu *et al.*, 2014 ▸). The averaged pulse energy after the monochromator is estimated to be 10 µJ with 100% shot-to-shot fluctuation because of the LCLS SASE characteristics. The pulse envelope duration is estimated to be about 40 fs FWHM on average as derived from the electron-bunch length in the undulator as well as the transverse cavity measurement (Behrens *et al.*, 2014 ▸).

We evaluated the timing overlap determination using four optical laser wavelengths, which were 1300 nm (outside of the CCD camera sensor sensitivity range), 800 nm (fundamental wavelength of the Ti:sapphire laser system), 520 nm (same wavelength with fluorescence from Ce:YAG) and 400 nm (the fluorescence of Ce:YAG can be induced by the optical laser excitation). The X-rays were focused with beryllium compound refractive lenses to 20 µm × 20 µm FWHM for the 520 nm and 1300 nm measurements, 15 µm × 15 µm FWHM for the 800 nm measurement and 50 µm × 50 µm for the 400 nm measurement, depending on the particular user experiment requirements during which the evaluations were made. These X-ray spot sizes are much smaller than the size of optical lasers used in typical pump–probe experiments.

**Figure 1:**
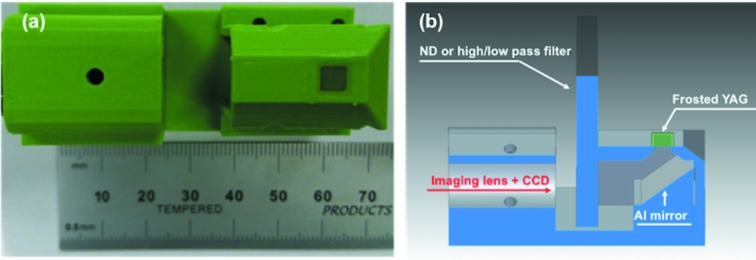
(*a*) Photograph of the t0-finder with a reference scale and (*b*) schematic layout of the t0-finder.

Fig. 1 ▸(*a*) shows a photograph of the described diagnostic which we term ‘t0-finder’ in short in this article and Fig. 1 ▸(*b*) shows the schematic layout of the setup. The t0-finder consists of a 3D printed frame, Ce:YAG screen (5 mm × 5 mm × 0.05 mm, Princeton Scientific Corp), aluminium-coated 45° prism and imaging optics (InfiniStix ×1 magnification compact lens, Infinity Photo-Optical Company). In order to improve the sensitivity to the optical laser transmission changes and to extract information only from the area where the X-ray and optical laser spots were overlapped on the sample surface, a roughened surface Ce:YAG scintillator with grounded surface (roughness is around 0.1 µm to 1 µm, typically called a ‘frosted YAG’) was utilized instead of a polished YAG screen. This enables the microscope to image the scattered optical laser light that reveals its profile on the surface, with and without fluorescence. We could thus optimize and achieve spatial overlap by monitoring the profile and position of both the X-ray and optical lasers with a µm resolution. The YAG thickness of 50 µm is almost matched to the penetration depth of 9.5 keV X-rays, but because of its diffusing surface only the optical laser scattering at a shallow depth close to the surface of the Ce:YAG is imaged on the 2D detector. Between the frosted Ce:YAG and the CCD camera (Adimec OPAL1000), there was a UV-enhanced aluminium-coated hypotenuse 45° prism to block residual X-rays and a neutral density (ND) filter (for 800 nm, 520 nm and 400 nm) or long-pass filter (Schott RG780) with a cut-off wavelength of 780 nm (for 1300 nm) to suppress the fluorescence background induced by X-rays and intense optical lasers and to enhance the signal-to-background ratio. The back surface of the frosted YAG was imaged on the CCD detector with a ×1 magnification optical lens, and its field of view and pixel size were 4.5 mm × 4.5 mm and 4.5 µm pixel^−1^, respectively, which could cover the entire screen of the Ce:YAG crystal, and had enough spatial resolution for the focused X-ray utilized for pump–probe measurements at XPP. The intensity of the optical laser was adjusted to be as high as possible by using a variable ND filter on the optical laser transport path to use the full dynamic range of the detector before saturation. This ND filter was also used to vary the laser energy for the later pump–probe experiments and thus contributed no offset to the measured t0. To vary the pump–probe delay in the measurements, we performed a step-by-step scan where the time delay is systematically scanned between discrete points and data is recorded at each point, and a fast scan stage where a fast delay stage moves continuously back and forth while recording the absolute optical delay (Glownia *et al.*, 2019 ▸). The pulse energy of each X-ray pulse was recorded by the beamline intensity position monitor (IPM) (Feng *et al.*, 2011 ▸; Tono *et al.*, 2011 ▸), and timing jitter between the optical laser pulses and X-ray pulses was compensated by using an arrival timing diagnostic, *i.e.* the ‘time tool’.

## 3. Results and discussions   

**Figure 2:**
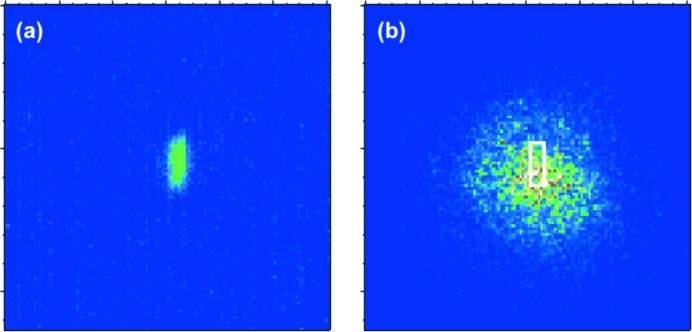
Averaged profiles on frosted Ce:YAG screen with 100 shots, (*a*) only X-rays and (*b*) X-ray and optical laser pulses. The white square shows a typical ROI to extract the t0 signal.

**Figure 3:**
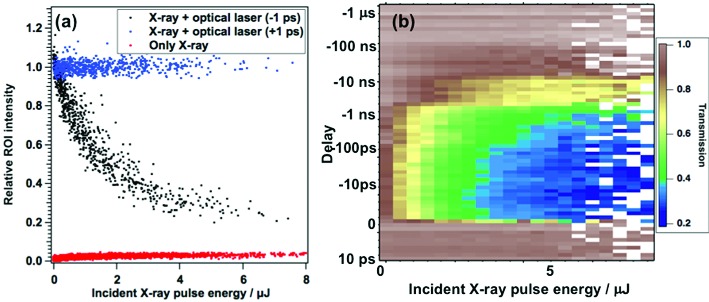
(*a*) Correlation plot between the integrated intensity of the ROI and the incident X-ray pulse energy, with red: only X-ray [focus diameter: 15 µm (FWHM)]; black: X-ray and optical laser (wavelength: 800 nm) at negative delay (−1 ps); and blue: X-ray and optical laser (wavelength: 800 nm) at positive delay (+1 ps). (*b*) Time delay and intensity dependences of the optical laser transmission (wavelength: 800 nm).

Fig. 2 ▸(*a*) is a typical averaged profile of focused X-rays and Fig. 2 ▸(*b*) is the profile with the optical laser (wavelength: 800 nm) on the frosted Ce:YAG of the t0-finder. We can see a speckly profile of the optical laser caused by the diffusing surface of the frosted YAG. To spatially resolve the overlap spot, we set a region of interest (ROI) around the focused X-ray profile, which was smaller than the optical laser profile. The integrated value of the ROI has clear positive correlation with the incident X-ray pulse energy [Fig. 3 ▸(*a*), red] and shows a saturated response. After achieving spatial overlap and minimizing the contribution from the fluorescence induced by the X-rays in the ROI, which is in positive correlation with the input X-ray pulse energy, the pulse energy of the optical laser was optimized by using the valuable ND filter so that there was either a negative correlation [Fig. 3 ▸(*a*), black] or no correlation [Fig. 3 ▸(*a*), blue] between the integrated intensity inside the ROI and the X-ray pulse energy (this correlation depends on the relative delay of the X-ray pulse to the optical laser pulse). The intensity ratio between the fluorescence induced by the X-rays and the optical laser was set to be 1:40–100 on the detector with a signal depth of 12 bit.

When the X-ray pulse arrived 1 ps earlier than the optical laser pulse, transmission of the optical laser within the ROI dropped down to <80% of the original level, which depends on the X-ray focus and pulse energy. A negative correlation between the ROI intensity and X-ray pulse energy was observed [Fig. 3 ▸(*a*), black]. On the other hand, no correlation was observed if the optical laser pulse arrived 1 ps earlier than the X-ray pulse [Fig. 3 ▸(*a*), blue]. Fig. 3 ▸(*b*) shows the intensity and time-delay dependences of the optical laser (800 nm) transmission from 10 ps to −10 µs. Negative delay means the X-ray pulse arrived before the optical laser pulse. X-rays can excite core electrons of the Ce:YAG and these excited electrons relax through a host of complex processes such as Auger decays, cascaded electron–electron scattering, self-absorption of its fluorescence and so on, which generates additional carriers in the conduction band and changes the Ce:YAG transmission of the optical laser. The excited charge carriers of Ce:YAG have on the order of up to several tens of nanosecond lifetimes and tails with microsecond scales (Zorenko *et al.*, 2005 ▸), which enable us to observe intensity dependence (negative correlation) up to a time delay of −1 µs. This allows us to find the temporal overlap between X-rays and optical lasers with up to a couple of 100 kHz operation and a time resolution of <100 fs starting from µs timescale temporal uncertainty by performing a simple binary search without having to measure rough timing first with a fast photodiode, which currently is a widely adopted first-step procedure for establishing pump–probe timing at all XFEL facilities. For XFELs with a higher repetition rate of >1 MHz, materials with shorter lifetimes or dropped shots to recover unpumped signal level are required.

**Figure 4:**
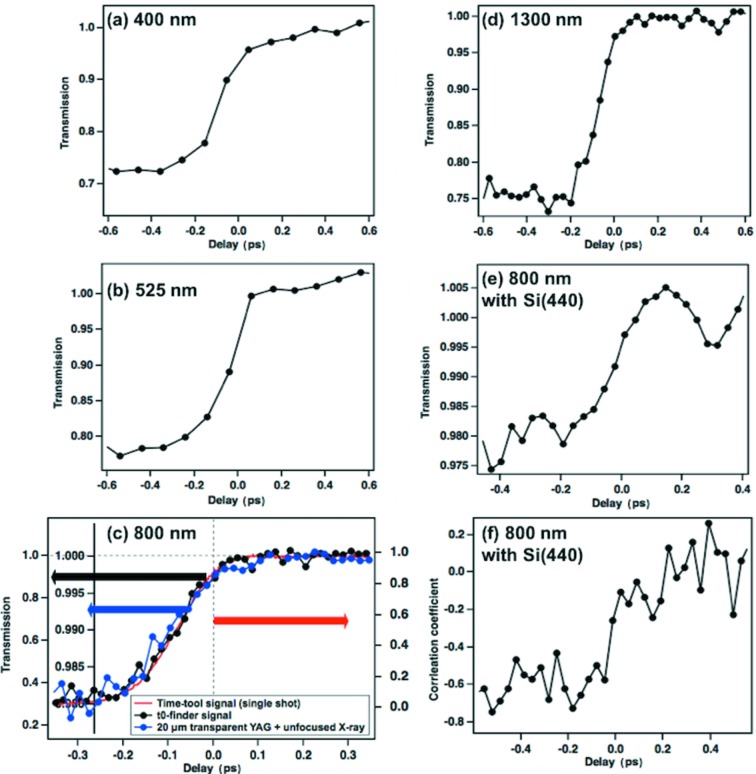
Transient optical transmission as a function of time delay of the laser with four different wavelengths: (*a*) 400 nm; (*b*) 520 nm; (*c*) 800 nm; red (right axis): single-shot signal of beamline timing monitor using 20 µm thickness with spectral encoding; blue (second-left axis): transient absorption signal using transparent 20 µm YAG, photodiode and unfocused X-ray; black (left axis): t0-finder signal; (*d*) 1300 nm; (*e*) 800 nm with monochromized X-rays after Si(440) monochromator; and (*f*) correlation coefficient as a function of time delay of 800 nm with monochromized X-rays after Si(440) monochromator. Both (*a*) and (*b*) are obtained by step-by-step scan with 100 fs step and 240 pulses at each delay position and sorted by time-tool signal for higher temporal resolution, while (*c*) and (*d*) are obtained by continuous scan with 120 s acquisition time for a total scan range from −2 to 2 ps, respectively, which are sorted by the time-tool signal.

We performed a transient absorption measurement with higher time resolution to determine temporal overlap with a resolution of <100 fs with four optical laser wavelengths. The resolution was improved in post-processing by using data from the beamline time tool [Figs. 4 ▸(*a*)–4(*d*)]. We observed that the transmission for each wavelength dropped down to 20%–80% without any normalization since the optical laser pulse energy is relatively stable compared with the SASE X-ray pulses after the monochromator.

We also compared the transient absorption signal of the t0-finder with the spectrally encoded single-shot signal of the beamline time tool with a 20 µm transparent Ce:YAG with pink beam [Fig. 4 ▸(*c*), red line], and the transient absorption signal obtained by scanning measurement with 20 µm of transparent YAG, photodiode, and unfocused monochromatic X-rays whose diameter was almost the same as the one with the optical laser [Fig. 4 ▸(*c*), blue]. The amplitudes of the measured transmission changes were different among these three signals, but the t0-finder signal [Fig. 4 ▸(*c*), black] reproduces the time response of the time-tool signal using the pink beam and the transient absorption signal obtained by a time-delay scan with 20 µm thickness of transparent Ce:YAG. The rising edge of the optical response of the t0-finder is calculated to be 160 fs (FWHM) by Gaussian fitting of the differentiated signal. By considering the thicknesses of 20 µm and 50 µm and the refractive index of Ce:YAG, the rising edge of material response was smeared to be 80 fs and 170 fs, respectively, because of the velocity mismatch between the optical laser and the X-ray pulses in addition to the convolution of their pulse widths. Sanchez-Gonzalez *et al.* (2017) ▸ reported a 1 ps scale degradation of the rising edge in the cross-correlation signal with 600 µm of Ce:YAG. However, there are no clear differences in the rising time behaviour in this comparison even though their thicknesses were different. This would be because (*a*) the detector of t0-finder was focused on the roughened surface and optical laser scattered by the surface and the contribution from the surface is large enough to change transmission of the bulk material, and because (*b*) the optical response of the Ce:YAG is limited by the cascade relaxation process with effective lifetime ∼160 fs. The exact time evolution of the band structures from deep ultraviolet to the visible region, relaxation process after core electron excitation, and the critical frequency of carriers after X-ray excitation are not straightforward to describe with a simple model, which should be the primary contributor to rising-edge degradation. The intrinsic 160 fs rising-edge width is unfortunately not accurate enough for precise determination of time zero, and it cannot be further deconvolved from the unknown optical response from first principles. However, the t0-finder signal with the fast scan can reproduce the rising edge and signal shape of the time-tool signal, which has an accuracy of better than 10 fs (RMS) (Mecseki *et al.*, 2018 ▸). A calibration of the response can be made by cross-checking the signal change level in the rising edge and the time zero with other known fast and characterized methods like time-resolved diffraction, streak measurements (Hartmann *et al.*, 2018 ▸) or other ultrafast material responses (Mecseki *et al.*, 2018 ▸). This cross-comparison allows us to resolve and identify the exact timing overlap in t0-finder signals with a temporal resolution of the time tool, ∼10 fs from the rising edge. In addition, the sensitivity of the measurement to only the surface enables us to use thicker crystals for this diagnostic while keeping good time resolution at the sample surface.

In order to evaluate the performance of the diagnostic with lower X-ray pulse energies and larger overall pulse energy fluctuations, we added an additional Si (440) channel-cut monochromator after the beamline monochromator. The bandwidth after the Si(440) was 0.09 eV and the throughput of the monochromator was ∼10%. Even though the transmission change induced by X-ray became smaller, we could still observe a clear time dependence without any normalization even though the X-rays have much larger intensity fluctuation after high-order reflection [Fig. 4 ▸(*e*)]. The high-fidelity measurement of the transmission signal changes, induced by the X-rays, has the capability to find timing overlap with each optical wavelength and higher-order X-ray monochromator without any normalization. However, in order to detect the signal change more sensitively with the smaller X-ray pulse energy and larger fluctuation we need to measure the correlation coefficient between the ROI intensity and the X-ray intensity instead of the integrated ROI intensity at each delay position. Here we take advantage of the fact that when the X-ray pulse comes first there should be a negative correlation. As can be seen in Fig. 4 ▸(*f*), the correlation coefficient changed from 0 (no correlation) to −0.8 at the negative delay (X-ray comes first) with a very low number of shots at each delay, while the averaged ROI intensity plot shows only 2% of the signal intensity dropping. This indicates that monitoring the correlation coefficient could potentially enhance the sensitivity of t0-finder for lower-flux cases with larger X-ray fluctuations.

## 4. Conclusion   

We developed a simple and robust diagnostic to determine spatial and temporal overlap between X-ray and optical laser pulses. Its performance was evaluated by using monochromatic X-ray pulses from LCLS and four different optical laser wavelengths. We demonstrated intensity and time-delay dependence of this diagnostic from femtosecond to microsecond time scales. The technique delivers reproducable signals and allows the determination of timing overlap down to tens of femtoseconds. The simplicity also makes it suitable for automatic beamline alignment for establishing spatial/temporal pump–probe experiments in the future.
